# Establishment of a Three-Dimensional In Vitro Model of Equine Papillomavirus Type 2 Infection

**DOI:** 10.3390/v13071404

**Published:** 2021-07-19

**Authors:** Anna Sophie Ramsauer, Garrett Louis Wachoski-Dark, Cornel Fraefel, Mathias Ackermann, Sabine Brandt, Paula Grest, Cameron Greig Knight, Claude Favrot, Kurt Tobler

**Affiliations:** 1 Institute of Virology, Vetsuisse Faculty, University of Zurich, 8057 Zurich, Switzerland; cornel.fraefel@vetvir.uzh.ch (C.F.); mathias.ackermann@vetvir.uzh.ch (M.A.); kurt.tobler@uzh.ch (K.T.); 2 Dermatology Unit, Vetsuisse Faculty, University of Zurich, 8057 Zurich, Switzerland; cfavrot@vetclinics.uzh.ch; 3 Internal Medicine, University Equine Clinic, University of Veterinary Medicine, 1210 Vienna, Austria; 4 Department of Veterinary Clinical and Diagnostic Sciences, Faculty of Veterinary Medicine, University of Calgary, Calgary, AB T2N 1N4, Canada; glwachos@ucalgary.ca (G.L.W.-D.); cgknight@ucalgary.ca (C.G.K.); 5 Research Group Oncology, University Equine Clinic, University of Veterinary Medicine, 1210 Vienna, Austria; Sabine.brandt@vetmeduni.ac.at; 6 Institute of Veterinary Pathology, Vetsuisse Faculty, University of Zurich, 8057 Zurich, Switzerland; grest@vetpath.uzh.ch

**Keywords:** horse, papillomavirus, EcPV2, 3D model, raft culture, squamous cell carcinoma, hyperplasia, plaque, skin

## Abstract

There is growing evidence that equine papillomavirus type 2 (EcPV2) infection is etiologically associated with the development of genital squamous cell carcinoma (SCC) and precursor lesions in equids. However, the precise mechanisms underlying neoplastic progression remain unknown. To allow the study of EcPV2-induced carcinogenesis, we aimed to establish a primary equine cell culture model of EcPV2 infection. Three-dimensional (3D) raft cultures were generated from equine penile perilesional skin, plaques and SCCs. Using histological, molecular biological and immunohistochemical methods, rafts versus corresponding natural tissue sections were compared with regard to morphology, presence of EcPV2 DNA, presence and location of EcPV2 gene transcripts and expression of epithelial, mesenchymal and tumor/proliferation markers. Raft cultures from perilesional skin harboring only a few EcPV2-positive (EcPV2+) cells accurately recapitulated the differentiation process of normal skin, whilst rafts from EcPV2+ penile plaques were structurally organized but showed early hyperplasia. Rafts from EcPV2+ SCCs exhibited pronounced hyperplasia and marked dysplasia. Raft levels of EcPV2 oncogene transcription (E6/E7) and expression of tumor/proliferation markers p53, Ki67 and MCM7 expression positively correlated with neoplastic progression, again reflecting the natural situation. Three-dimensional raft cultures accurately reflected major features of corresponding ex vivo material, thus constituting a valuable new research model to study EcPV2-induced carcinogenesis.

## 1. Introduction

Papillomaviruses (PVs) belong to a family of small mucocutaneous viruses that can induce benign disease or cancer in humans and a wide range of animals [1]. PVs consist of a non-enveloped capsid enclosing a relatively short (~8 kbp), circular dsDNA genome consisting of an early region (E), a late region (L) and a non-coding long control region (LCR). The E region comprises open reading frames (ORFs) encoding for regulatory proteins such as E1, E2 and E4, and transforming proteins such as E6, E7 and frequently E5. The late (L) region encodes the viral capsid proteins L1 and L2. The LCR that is located between the L1 and E6 ORFs regulates viral replication and transcription [1].

Early research in animal models has revealed the high species-specificity of PVs and their usual tropism for keratinocytes [2]. Importantly, PVs have evolved a remarkable replication strategy that is tightly linked to the differentiation program of keratinocytes [3]. De novo infection by PV virions occurs via micro-abrasions that give access to basal keratinocytes, which present the necessary surface molecules for virion attachment and endocytosis [4,5,6]. Following virion disassembly, early viral regulatory and transforming proteins are expressed in the basal and suprabasal epidermal layers [3]. In the differentiated cells of the spinous and granular layers, the viral genome replicates to 10–100 copies per cell. The granular and final squamous layers provide the ideal cellular environment for capsid protein expression and directed virion assembly. Finally, new infectious virions are shed via desquamation [7].

PV infection is not always productive; however, the viral genome may be maintained in infected cells as multiple episomes that replicate in synchrony with the cell cycle [8] or even integrate into the host cell genome [9].

To date, more than 200 different human PV (HPV) types have been identified (https://pave.niaid.nih.gov, accessed on 1 June 2021) [10]. Of these, 15 types have been designated as high-risk HPVs (hrHPVs) (https://pave.niaid.nih.gov, accessed on 1 June 2021) [10] because of their etiological association with virtually 100% of cervical cancers, at least 50% of anogenital squamous cell carcinomas (SCCs) and about 25% of head and neck SCCs [11].

SCCs also occur in horses and other equids, where they account for up to 37% of all tumors [12]. Equine SCCs can develop at any site on the integument or other stratified squamous epithelium, yet they preferentially affect mucocutaneous junctions, i.e., the (peri-)ocular and genital regions [13]. Usually, the initial stages of genital SCCs present as whitish plaques or papillomas that can progress to carcinoma in situ (CIS) and ultimately to SCC [12].

Whilst the etiology of equine (peri-)ocular SCCs is still unknown; there is growing evidence that equine papillomavirus type 2 (EcPV2) infection is causally associated with the development of genital SCCs and precursor lesions. This evidence is mainly based on the consistent detection of EcPV2 DNA and transcripts in up to 100% of these lesions and on the low prevalence of EcPV2 infection in asymptomatic horses [14,15,16,17,18,19,20]. Interestingly, EcPV2 DNA and mRNA have also been found in a subset of equine oropharyngeal SCCs, gastric SCCs and their metastases [19,21,22,23]. The concept of a causal association between EcPV2 infection and equine genital tumors and a subset of oropharyngeal SCCs is further supported by the observation that EcPV2 infection can be productive in these lesions and that EcPV2 DNA can integrate into the genome of infected tumor cells [19,20]. However, the exact pathobiological mechanisms underlying the postulated EcPV2-induced development of equine SCCs remain to be elucidated.

The dependence of the PV lifecycle on the differentiation program of keratinocytes has always been an obstacle to the establishment of in vitro models of PV infection. This obstacle was overcome with the advent of organotypic raft culture systems that faithfully recapitulate epithelial differentiation and thus provides a permissive environment for completion of the PV life cycle [24]. Here we describe the establishment and basic evaluation of the first three-dimensional (3D) raft culture model of EcPV2-infected equine penile perilesional skin, plaques and SCC.

## 2. Materials and Methods

### 2.1. Sample Material

Skin tissue samples were collected from three different equine patients with penile lesions, which underwent partial phallectomy for therapeutic reasons in 2017 and 2018 at the equine clinics of the Vetsuisse Faculty of the Universities of Zurich and Berne, Switzerland. The three patients were (i) a 15-year-old Lusitano gelding affected by penile SCC and plaques, (ii) a 17-year-old Icelandic gelding with recurrent penile SCCs and papillomas and (iii) an 18-year-old Swiss Warmblood gelding displaying a penile papilloma as well as plaques on the penis and the prepuce. Tissue samples of lesional (SCC and/or plaques) and perilesional skin were collected. Three samples were collected from each site; one was transferred to a transport medium [25] for keratinocyte isolation, one was frozen and stored at −20 °C for downstream molecular biological analyses, and one was fixed in 4% paraformaldehyde, embedded in paraffin and subjected to H&E staining for histological analysis.

### 2.2. Isolation of Keratinocytes

Primary equine keratinocytes were isolated from penile SCCs, plaques and perilesional skin (SCC margins) using conditioned reprogramming as described previously [25,26,27] with slight modifications. In brief, tissue samples in transport medium [25] were washed extensively using miconazole and chlorhexidine-containing shampoo (Malaseb^®^, Dechra Pharmaceuticals PLC, Dornbirn, Austria), rinsed with 100% ethanol and then rinsed twice with PBS. The tissue samples were incubated for 24 h at 4 °C in dispase (STEMCELLS Technologies Germany GmbH, Cologne, Germany) and complete F medium [25] (1:1) to separate the epidermis from the dermis. The epidermal layers were peeled from the tissue samples, mechanically disrupted using a scalpel and incubated in a 1:1 mixture of trypsin (0.25%; Thermo Fisher Scientific, Basel, Switzerland) and DMEM (Sigma-Aldrich, Merck & Cie, Schaffhausen, Switzerland; without FCS) on a rocking platform for 10–30 min to obtain single cells. Following trypsin inactivation by addition of complete DMEM (containing 10% FCS) [25], cell suspensions were filtered using a 100 µm cell strainer (Sigma-Aldrich, Merck & Cie, Schaffhausen, Switzerland), centrifuged for 10 min at 500× *g* and resuspended in a complete F medium containing 10 mM Rho-associated coiled-coil-containing protein kinase (ROCK) inhibitor Y-27632 (Y) (STEMCELLS Technologies, Germany GmbH, Cologne, Germany). Cells were seeded in flasks containing irradiated (30 Gy) Swiss-3T3-J2 mouse fibroblasts (IRJ2) at 50–70% confluency as feeder cells and incubated at 37 °C and 5% CO_2_ [25]. The medium was changed every other day, and cells were split at 70–90% confluency.

### 2.3. EcPV2- and ecGAPDH-PCR

To confirm their equine origin and to screen for EcPV2 infection, proliferating early-passage cells were subjected to DNA extraction using QIAamp DNA Minikit (Qiagen, Hombrechtikon, Switzerland) according to the manufacturer’s instructions. PCR reactions were performed from 1 μL DNA aliquots using (i) horse specific GAPDH primers (ecGAPDHi forward primer: 5′-ATC CGG AGT CTT CCA CTC CA-3′, ecGAPDHi reverse primer: 5′-GTC GGA GGG TTA ACC ACA GG-3′) and (ii) EcPV2-specific primers for amplification of a 395 bp region within the E6 gene (18). Reaction mixtures of 25 μL were prepared according to the manufacturer’s instructions (REDTaq ReadyMIX, PCR Reaction mix; Sigma-Aldrich, Merck & Cie, Schaffhausen, Switzerland) and then subjected to a denaturation step of 94 °C for 3 min, followed by 40 amplification cycles of 94 °C for 30 s, 55 °C for 30 s and 72 °C for 30 s per cycle. Amplicons were separated by 1% TAE-gel electrophoresis and visualized by GelRed^®^ Nucleic Acid Gel Staining (Biotium, Inc. Fremont, CA, USA).

### 2.4. Three-Dimensional (3D) Air-Liquid Interface Culture

Approximately 300,000 highly proliferating cells were seeded with IRJ2 in 12 mm 0.4 µm pore size polycarbonate cell culture inserts (Millipore PIHP011250, Merck & Cie, Schaffhausen, Switzerland) in a 24 well plate containing F + Y medium inside and outside the insert at equal levels. The medium was changed on the next day and substituted on day 3 with FTAC medium (CellnTec, Berne, Switzerland) containing 10mM Y-27632 (Y) to allow for the formation of intercellular adhesions. On day 4, airlift was performed, where the medium was removed, and inserts were transferred to six-well plates (one insert per well), each well containing 2 mL FTAC + Y medium surrounding the insert to the membrane level. The medium was changed every other day. The cells were grown for 12 more days on air-liquid-interface to 3D rafts. The rafts were collected, fixed in 4% paraformaldehyde, embedded in paraffin and subjected to H&E staining. Three representative rafts and corresponding tissue material were sectioned for immunohistochemistry (IHC) and EcPV2-specific RNA in situ hybridization (RISH).

### 2.5. EcPV2 RNA In Situ Hybridization

To assess tissue and derived raft sections for EcPV2 oncogene transcription, RISH was carried out on three representative FFPE tissue and raft sections as described previously [28]. In brief, a multiple-probe chromogenic RISH method (RNAscope) designed to hybridize to EcPV2 E6/E7 [29] was run on five replicates per lesion or raft using an RNAscope 2.5 HD Detection Kit RED (ACDbio; Newark, CA, USA) according to the manufacturer’s instructions with minor modifications. Instead of xylene, Xylene Substitute (Sigma-Aldrich) was used; to expose nucleic acids, slides were placed into boiling 1× target retrieval solution for 15 min, and counterstaining was performed in 20% instead of 50% hematoxylin.

### 2.6. Immunohistochemistry for Epithelial, Mesenchymal, Cancer and Proliferation Markers

Unstained paraffin-embedded tissues were sectioned consecutively at 3.5 μm thickness and placed onto positively charged glass slides. IHC staining was performed using an automated staining device (Dako Autostainer, Dako, Agilent Technologies AG, Basel, Switzerland) with diaminobenzidine (DAB) or 3-amino-9-ethylcarbazole (AEC) (Dako, Agilent, Santa Clara, CA, USA) serving as chromogen and Meyer’s hematoxylin as counterstain. Negative controls were prepared by omitting the primary antibody, while normal equine skin, SCC tissue and a lymph node served as positive controls. Detailed incubation conditions and respective providers for the antibodies used (anti-vimentin, -pan-cytokeratin (PCK), -p53, -Ki67 and -MCM7) are given in Table 1.

## 3. Results

### 3.1. Sections from 3D Rafts and Corresponding Ex Vivo Tissue Material Exhibited Similar Histomorphological Features

Primary equine keratinocytes from penile SCCs, penile plaques and/or perilesional skin from penile tumor margins of three horses were isolated and expanded in cell culture. DNA isolated from original tissue and derived cell culture aliquots tested positive by equine GAPDH PCR, thus confirming the PCR suitability and equine origin of the cultured cells. All isolates were positive for EcPV2 DNA.

**Figure 1:**
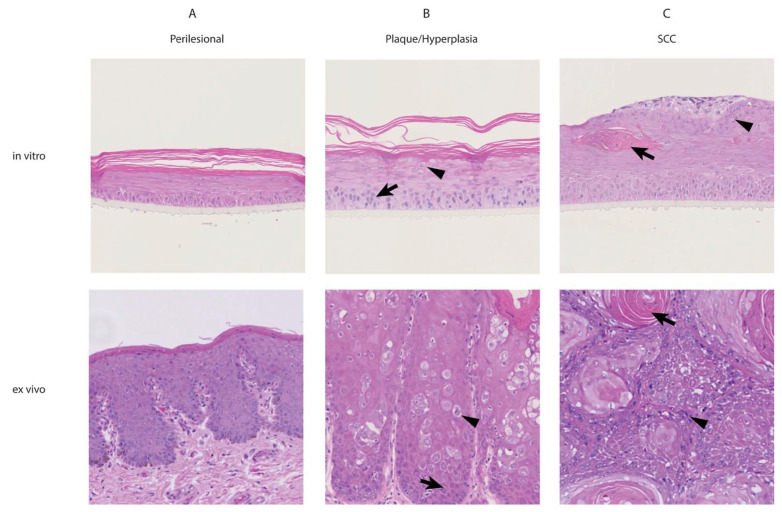
H&E staining of raft cultures and corresponding tissue sections. Representative areas of perilesional (**A**), plaque/hyperplasia (**B**) and squamous cell carcinoma (SCC) (**C**) raft cultures and tissue sections. Raft cultures derived from the three different ex vivo tissue sources correspond to their respective tissue of origin in terms of proliferation and differentiation. (**A**) Raft cultures derived from perilesional skin accurately reflect the keratinocyte differentiation process of normal skin. (**B**) Raft cultures derived from penile plaques are structurally organized but show early hyperplasia and crowding of basal cells (arrow) and hypertrophy of keratinocytes with occasional perinuclear halos (arrowhead). This is similar to the foci of hyperplasia (arrow) and koilocyte-like cells (arrowhead) present in the corresponding tissue section. (**C**) Raft cultures derived from penile SCCs exhibit marked hyperplasia and dysplasia. Of note are clusters of anucleate eosinophilic cells, suggestive of early keratinization (arrow), and clusters of basaloid cells (arrowhead) within the stratum spinosum, which likely mimic the keratin pearls (arrow) and infiltrative islands of epithelial cells (arrowhead) that characterize SCCs. All photographs taken using 10× objective. H&E stain.

Cells were cultured on an air-liquid interface and grown to 3D rafts. Major histomorphological features of the raft and corresponding tissue sections were determined by H&E staining (Figure 1). Raft cultures established from perilesional penile skin were clearly organized into a basal, a spinous and a cornified cell layer. A granular layer could not be identified unequivocally in all sections by microscopy—basal cells presented as a single row of columnar cells containing oval, frequently apical nuclei. The spinous layer was composed of three to five rows of differentiating polygonal cells. The comparatively thick cornified layer consisted of fully differentiated, anucleate keratinocytes arranged in a compact basket-weave pattern. When comparing the overall organization of these perilesional skin-derived raft cultures to sections of source material, a high degree of similarity regarding cell differentiation was noted (Figure 1A).

Raft cultures established from EcPV2-positive penile plaques histologically classified as benign hyperplasia recapitulated a precancerous stage. They were thicker than raft cultures derived from perilesional skin, and the basal layer exhibited a crowded structural pattern, with one to three layers of basaloid cells containing few mitotic figures or apoptotic cells. The stratum spinosum consisted of a maximum of eight rows of normally differentiating keratinocytes—The stratum corneum did not differ from the stratum corneum of the raft culture derived from perilesional skin. Overall, 3D cultures established from equine penile plaques authentically reflected the histomorphological characteristics of the original ex vivo tissue, with acanthosis (thickening) of the epithelium and multifocal hypertrophy of keratinocytes, suggesting koilocyte-like cells formation (Figure 1B).

Raft cultures established from penile SCC tissue were characterized by pronounced thickening and disorganization of all cell layers. The basal layer exhibited a crowded structural pattern, with basaloid cells growing in more than one direction in one to three superposed rows. In addition, mitotic figures and apoptotic cells were identified. The stratum spinosum presented as a pronouncedly thick layer of at least seven rows of differentiating keratinocytes. Although differentiation appeared morphologically normal, growth direction was not always conserved. In addition, areas of cellular degeneration with nuclear debris in vacuolated cells were seen, along with foci of anucleate eosinophilic cells or cell clusters, suggestive of early keratinization. Clusters of basaloid cells were also identified in areas of apparently normal differentiation in the upper part of the spinous layer. The stratum corneum presented as an unusually thin layer. Interestingly, it contained clusters of basaloid cells, thus modeling the in vivo situation for equine penile SCCs, where islands and trabeculae of epithelial cells with or without keratinization infiltrate the underlying dermis (Figure 1C).

### 3.2. Equine Penile Lesions and Derived Raft Cultures Contained Multiple Copies of EcPV2 Nucleic Acids

**Figure 2:**
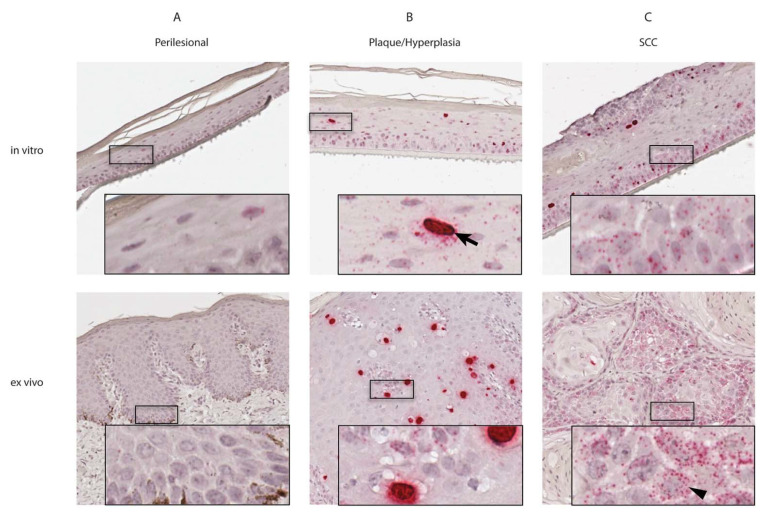
Equine papillomavirus type 2 (EcPV2) signal distribution patterns in raft cultures and corresponding tissue sections using RNA in situ hybridization (RISH). EcPV2 RISH of representative areas of perilesional (**A**), plaque/hyperplasia (**B**) and squamous cell carcinoma (SCC) (**C**) raft cultures and tissue sections. (**A**) Perilesional skin-derived rafts and tissue sections contain just a few cells with weak granular signal (GS). (**B**) In plaque-derived rafts and tissue sections, moderate GS and scattered diffuse nuclear signal (DNS) (arrow) is present. (**C**) In SCC-derived rafts and tissue sections, strong GS (arrowhead) is present, while rafts also display rare DNS. All photographs taken using 10× objective.

To determine the distribution pattern of EcPV2-infected cells within ex vivo material and derived raft cultures, corresponding sections were subjected to EcPV2 E6/E7-specific RNA in situ hybridizations (RISH). Both lesional tissue and corresponding raft cultures were shown to harbor multiple copies of EcPV2 nucleic acids. In addition, RISH yielded two distinct signal patterns: (i) a finely scattered granular signal (GS) throughout the nucleus and cytoplasm of infected cells or (ii) an intense diffuse nuclear signal (DNS) filling the entire nucleus (Table 2 and Figure 2).

In general, perilesional skin-derived raft sections were negative by RISH, with only a few single cells displaying a weak GS. Corresponding tissue sections presented the same picture, with only a few cells in the upper layers of the immediate tumor margins exhibiting a weak GS (Figure 2A).

Plaque-derived raft sections displayed moderate GS throughout the basal and the spinous layers. Occasional cells in the spinous layer exhibited DNS. Similarly, penile plaque tissue sections displayed a moderate GS in the majority of cells, while GS was more pronounced in basal and suprabasal epidermal layers. Many cells, especially those in the upper layers, exhibited strong DNS (Figure 2B).

In SCC-derived rafts, a strong GS was detectable in all basal cells and in clusters of basaloid cells in the upper keratinocyte layers. The vast majority of cells in the more differentiated layers exhibited a weaker GS or were negative. DNS was present in a few cells within the stratum basale, stratum spinosum and basaloid cell clusters in upper layers (Figure 2C). This might reflect the infiltrating epithelial islands characteristic of SCC tissue sections, which also presented a predominantly strong GS in the majority of cells in basal and upper layers, while more differentiated cells displayed weak to no GS. DNS was displayed by only a few individual cells (Figure 2C).

### 3.3. Lesional Raft Cultures Exhibited Enhanced Expression of Tumor and Proliferation Markers

**Figure A1:**
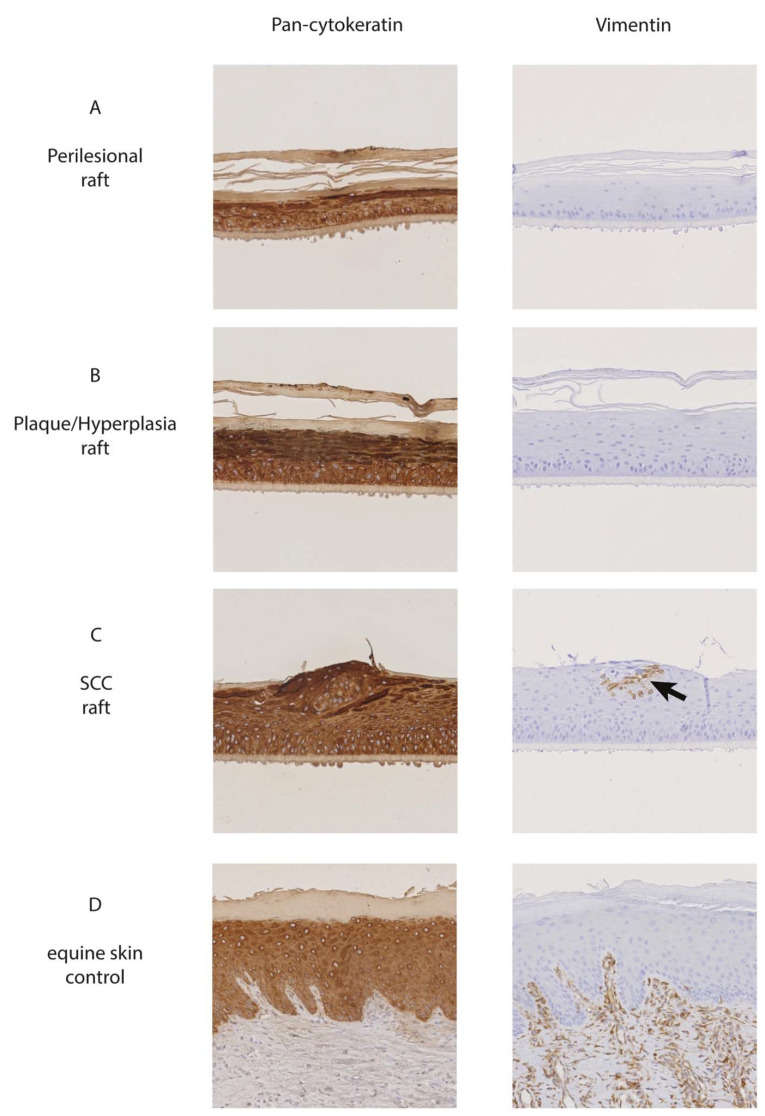
Pan-cytokeratin and vimentin immunostaining of raft cultures. Pan-cytokeratin and vimentin immunostaining of representative areas of perilesional skin-derived (**A**), plaque/hyperplasia-derived (**B**), squamous cell carcinoma (SCC)-derived (**C**), raft cultures and equine skin as control (**D**). All cells of the raft cultures are positive for the epithelial marker pan-cytokeratin, while the mesenchymal marker vimentin is not expressed within the raft cultures, except in a few cells within basaloid clusters in the upper layers of the SCC-derived raft ((**C**)—arrow). The equine skin control shows pan-cytokeratin positive epithelial cells in the epidermis and vimentin-positive mesenchymal cells in the dermis. No vimentin-positive cells are present in the epidermis. All photographs taken using 10× objective.

Tissue sections and derived raft cultures were analyzed by IHC. First, tissue and raft culture sections were tested for expression of the epithelial cell marker pan-cytokeratin and the mesenchymal cell marker vimentin to confirm the epithelial origin of the rafts. In all tissue sections, the epidermal parts of the skin, including infiltrative islands of cells present in SCCs, tested positive by pan-cytokeratin staining. In contrast, the dermis and, very rarely, lesional epidermal tissue tested positive by vimentin staining. A representative staining control of equine skin is shown in Figure A1. Independent of their tissue of origin, all raft culture sections likewise tested positive for pan-cytokeratin throughout all cell layers. Raft culture sections derived from perilesional skin or penile plaques tested negative for vimentin expression. In contrast, SCC-derived raft culture sections contained sporadic vimentin-expressing cells in upper epithelial layers, in particular within clusters of basaloid cells (Figure A1).

**Figure 3:**
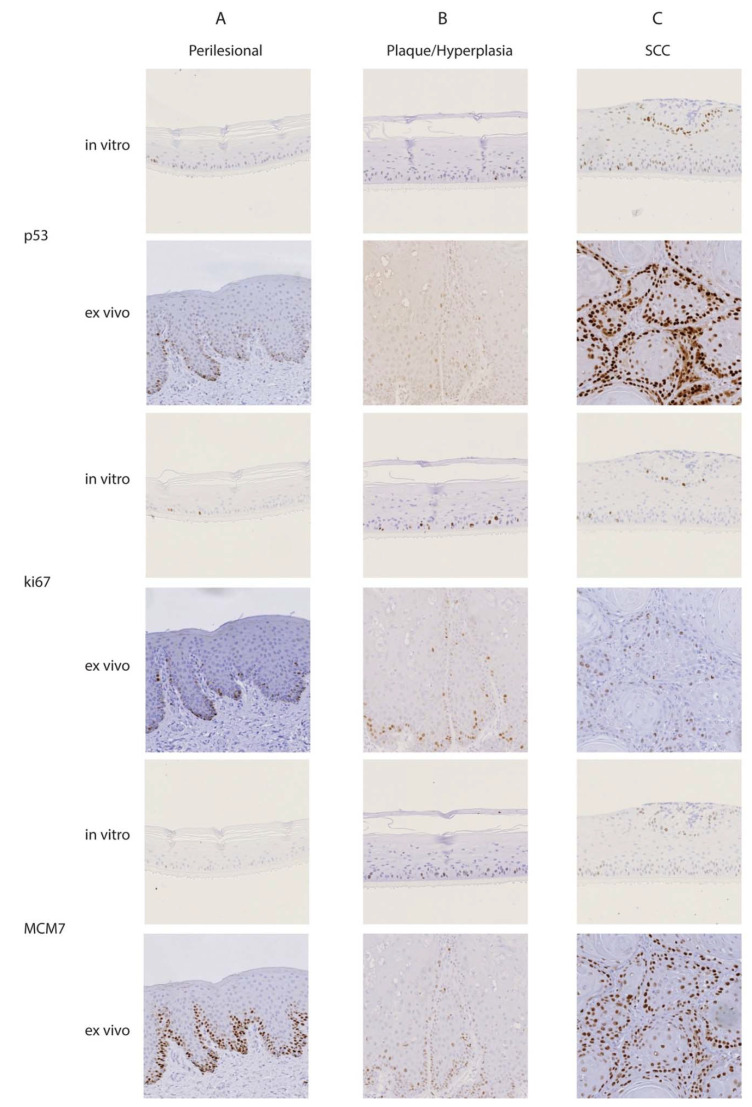
p53, Ki67 and MCM7 immunostaining of raft cultures and corresponding tissue sections. Immunostaining using p53, Ki67 and MCM7 antibodies of representative areas of perilesional (**A**), plaque/hyperplasia (**B**) and squamous cell carcinoma (SCC) (**C**) raft cultures and tissue sections. Expression of p53, Ki67 and MCM7 increases with increasing neoplastic phenotype within both rafts and tissue sections of EcPV2-associated lesions. All photographs taken using 10× objective.

Rafts and corresponding tissue sections were also evaluated for expression of the tumor marker p53 and of the proliferation markers Ki67 and MCM7 (Figure 3). 

In rafts derived from perilesional skin, only a few basal cells (<10%) were positive for p53, Ki67 and MCM7, and signals were restricted to the basal layer. Perilesional skin sections showed a similar staining distribution, with MCM7 staining being more pronounced in tissue than in raft sections (Figure 3A).

In plaque-derived rafts, a moderate number (10–50%) of basal cells, as well as suprabasal cells located in proximity to the basal membrane, expressed p53, Ki67 and MCM7. Approximately 50% of basal and suprabasal cells stained positive for these markers in plaque tissue sections (Figure 3B).

In SCC-derived rafts, a high number (>50%) of basal and suprabasal cells scored positive for p53. Importantly, this protein was also expressed in upper cell layers. The expression of p53 was particularly high (>90%) within clusters of basaloid cells, whereas the more differentiated cells within these clusters were negative. In SCC tissue sections, staining of infiltrating islands of keratinocytes showed the same p53 expression pattern. Similarly, basaloid cell clusters observed in SCC-derived rafts and infiltrating islands of keratinocytes identified in SCC tissue sections both exhibited moderate Ki67 and high MCM7 expression, whilst more differentiated cells within these clusters or islands were negative for these markers (Figure 3C).

## 4. Discussion

There is growing evidence that EcPV2 is actively involved in the pathogenesis of equine genital SCCs via the combined transforming activity of the two viral oncoproteins E6 and E7 [18]. However, the exact mechanisms underlying EcPV2 oncoprotein-induced carcinogenesis have not been studied so far. This is in part due to the lack of in vivo and in vitro models accurately reflecting EcPV2 infection and associated disease. To overcome this significant limitation, we aimed to establish an in vitro model for EcPV2-associated equine genital lesions. To ensure maximum biological authenticity and allow for comparison between low-grade and high-grade lesions, we chose to generate three-dimensional raft cultures from fresh perilesional skin (tumor margins), benign SCC-precursor lesions (i.e., penile plaques) and SCC of the penis.

Following confirmation of EcPV2 infection in ex vivo tissues and derived 2D cell cultures, cells were subsequently grown in 3D. Then sections from obtained rafts and their respective tissue source were comparatively analyzed as to their histomorphological features. Importantly, all rafts authentically reflected their respective biological source with regard to the overall structural and cellular organization [28]. Three-dimensional raft culture allowed keratinization of equine keratinocytes during terminal differentiation into cells of the stratum corneum as observed in vivo [30]. Rafts established from penile perilesional skin displayed the typical multi-layered architecture of normal epidermis. However, the stratum granulosum could not be unequivocally identified in all raft and corresponding tissue sections by light microscopy, meaning that we could only presume the presence of this layer within all rafts. Analysis of sections by electron microscopy or by IHC using stratum granulosum-specific markers such as loricrin and/or filaggrin might help to confirm this presumption [31,32].

The high histomorphological similarity between perilesional skin tissue derived rafts and cultures established from normal equine skin [33] was indicative of low viral activity. This concept is also supported by RISH detecting only a few EcPV2-infected cells in perilesional tissue and derived raft sections. In contrast, lesional raft sections contained large numbers of infected keratinocytes. Consistent with this observation, moderate (plaque-derived rafts) to substantial (SCC-derived rafts) histomorphological changes were noted, supporting the concept that EcPV2 has an active role in disease onset and progression. Analogous changes were also detected in corresponding ex vivo material from which rafts were established: EcPV2-infected plaques displayed thickening of the epithelium with orderly maturation along with crowding of basal cells and the frequent presence of koilocyte-like cells, as reported previously [28]. Plaque tissue sections also exhibited the formation of broad rete ridges—A feature that cannot be recapitulated in this 3D culture system. Similar results have been reported for raft cultures established from an HPV16-transfected isogenic keratinocyte cell line (NIKS) reflecting low-grade squamous intraepithelial lesions (SIL) [34], and in studies of HPV31-transfected primary human foreskin cells [24,35]: HPV16 and HPV31-transfected rafts were thicker than rafts established from non-infected cells, reflecting increased cell proliferation rates and the ability of these HPV types to maintain differentiating cells in an active cell cycle phase [24,34,35]. Equine penile SCC-derived rafts resembled HPV16 transfected NIKS rafts mimicking high-grade SIL lesions with respect to increased thickness, higher cell proliferation rates and a higher degree of dysplasia in comparison to low-grade SIL [34]. 

Abnormal differentiation in equine penile SCC-derived rafts was characterized by clusters of basaloid cells in upper, usually differentiated cell layers and by keratinized cells in intermediate, typically non-cornified, layers. These clusters likely recapitulate islands of neoplastic epithelial cells (with or without keratinization) that characteristically infiltrate the underlying dermis in EcPV2-associated penile SCCs [28]. Full-thickness skin models mimicking epidermis and dermis are used in hrHPV-associated cancer research to study invasion mechanisms and treatment options [36,37]. The in vitro model we describe here consists only of epidermis and so cannot mimic invasive tumor growth. Therefore, our future studies will focus on the development of full-thickness raft cultures that also include a dermal layer.

Plaque and SCC-derived rafts contained multiple copies of EcPV2 E6/E7 mRNA as determined by RISH, indicating active transcription of the viral oncogenes. Two distinct signal patterns—granular signal (GS) and diffuse nuclear signal (DNS) (Figure 2)—were present. This dual signal pattern has been previously described for equine EcPV2-associated genital lesions [28,29] and HPV16-associated cervical intraepithelial neoplasms (CIN) [38]. GS reflects the presence of probe-specific mRNA, while DNS is believed to result from hybridization with both mRNA and the “unzipped” viral ssDNA that emerges during PV replication [29]. Thus, the term RISH is somewhat of a misnomer in that the technique detects both PV RNA and DNA. The GS was more pronounced in basal and suprabasal cells, while less hybridization was noted in more differentiated cells in the upper layers of lesional tissue and rafts. This is likely due to the fact that in these cells, regulatory E2 protein downregulates E6 and E7 expression to allow for cell differentiation and entrance into the differentiation-dependent stage of the viral life cycle, as shown for HPV16 [39]. Interestingly, DNS is reported to be far more pronounced in benign hrHPV and EcPV2-induced lesions than in malignant lesions, in which DNS was minimal or absent [28,38]. This apparent paradox is likely due to the reduction in PV replication that occurs as the PV genome integrates into the host genome and productive infection ceases. Since PV integration is associated with increased malignancy, the absence of DNS in a PV-induced lesion may suggest a more aggressive lesion. Correspondingly, the presence of abundant DNS in a PV-induced lesion may be associated with a better prognosis [38].

No DNS but occasional weak GS was present in rafts from tumor margins, suggesting low viral infection and transcription levels in the tumor periphery. This observation is in agreement with the first EcPV2 E6 in situ hybridization report describing a “demarcation line” between EcPV2-positive tumor tissue and non-infected normal tissue surrounding the lesion [17], and a recent report showing GS and DNS exclusively within equine penile lesions [28]. In addition, the lack of DNS in perilesional rafts testifies for the accuracy and specificity of the assay.

In rafts established from benign penile plaques (benign hyperplasia), the majority of cells had a moderate GS, which was more pronounced in basal and suprabasal epidermal layers than in higher layers. DNS was predominantly detected in spinous layers. These observations were in accordance with signal patterns in plaque tissue sections, thus confirming previous data [28]. Our data also agree with the finding that hrHPV E6/E7 expression induces hyperproliferation and cell cycle entry in basal and suprabasal human epithelial layers and drives cell cycle entry in suprabasal keratinocytes to allow for viral genome amplification [9]. Therefore, we presume that EcPV2 E6 and E7 also drive hyperproliferation of basal keratinocytes as reflected by the abnormal thickness of the basal layer. In upper more-differentiated layers, we further propose that DNS within the stratum spinosum of plaque-derived rafts is indicative of high EcPV2 genome replication rates.

SCC-derived rafts displayed a similar signal pattern to that of plaque-derived rafts, however, the GS was significantly more pronounced. In addition, clusters of basaloid cells detected in upper keratinocyte layers displayed GS and DNS, indicating an influence of EcPV2 on their cell differentiation and proliferation processes. In accordance with this observation, analysis of SCC tissue sections revealed strong GS within islands of cells infiltrating the dermis. We propose that the clusters of basaloid cells in the upper layers of the raft culture model represent the infiltrating islands characteristic of SCC tissues that result from virally induced perturbation of cell proliferation. Stronger GS observed in SCC tissue and rafts as compared to plaque tissue, and rafts indicate enhanced oncogene expression and transforming activity. In human cervical lesions, oncogene expression levels correlate with disease progression, and also with disruption of the normal productive lifecycle (9). Enhanced E6 and E7 expression may also be due to integration events, which are usually accompanied by disruption of the E2 ORF and hence loss of this protein’s function as a regulator of oncogene expression [9]. This assumption is substantiated by previous findings that EcPV2 can integrate into the host cell genome [19,20]. However, DNS displayed by lesional raft sections, which was only rarely present in SCC tissues, suggests that EcPV2 DNA replication occurs within the rafts. Nevertheless, the unambiguous demonstration of virion assembly in raft cultures has not been successful so far. As the DNS pattern is only sporadically seen in SCC tissue but is consistently present in SCC-derived rafts, we may assume that the bare process of culturing those cells on rafts is capable of reactivating dormant EcPV2 replication. External stress factors may cause the same in vivo, thus, securing EcPV2 survival in infected tissues and providing time for a staggering evolution towards malignancy of the lesion.

The epithelial origin of the rafts was confirmed by the expression of the epithelial marker pan-cytokeratin and the absence of the mesenchymal marker vimentin. Interestingly, in a few cells within SCC-derived rafts, there was detectable vimentin immunostaining. As this was seen in proliferating areas within upper layers of the SCC-derived rafts, this could be due to the invasive and migratory potential within these cells, potentially undergoing epithelial to mesenchymal transition (EMT), as already described in cervical carcinoma rafts [40]. This interesting finding potentially pointing to the presence of EMT in equine SCCs might be expected, given the metastatic potential of SCCs in general and of EcPV2-associated penile SCC in particular [12]. Our 3D skin model could possibly serve as an in vitro study of EMT in EcPV2. As EMT is a crucial step in malignant tumor progression, this would be an additional achievement. However, vimentin expression in various cultured epithelial cells has also been described previously and interpreted as being an artifact induced through the in vitro cultivation process [41,42]. Furthermore, since the culture conditions contained irradiated murine fibroblasts, the possibility of contamination by fibroblasts cannot be fully excluded. However, this would not explain why vimentin-positive cells were only present in SCC-derived rafts in our work. Further studies are needed to address this topic.

Expression of the tumor marker p53 and the proliferation markers ki67 and MCM7 directly correlated with EcPV2 oncogene transcription levels and neoplastic progression ex vivo and in vitro. Of note, high p53, ki67 and MCM7 expression was particularly exhibited by basaloid cell islands within upper epithelial layers, as previously described for equine penile tumor sections [28]. Higher expression of proliferation markers has also been reported for hrHPV raft culture models [34,43]. In human cervical lesions, ki67 overexpression is associated with severity and progression of cervical neoplasia, while the data regarding p53 expression are controversial [44].

In hrHPV infection, E7 triggers the uncontrolled S-phase entry, which in turn leads to p53 tumor suppression activation [45]. The effect of p53 expression is abrogated by E6-mediated degradation of this tumor suppressor [46]. Alpha genus PV E6 proteins bind to the LXXLL peptide of E6AP, a cellular ubiquitin ligase, and form a ternary complex with p53, thereby stimulating the ubiquitin ligase activity promoting p53 degradation [47,48]. Most other PV E6 proteins bind to a similar LXXLL peptide on the cellular transcriptional co-activator MAML1, thereby repressing Notch signaling and altering differentiation [48]. The E6 proteins display only a low degree of conservation, although the EcPV2 E6 protein sequence clusters closer to the Alpha genus HPV E6 proteins than to the other HPV genera [48]. However, as no structural data exist for EcPV2 so far, no conclusions can be drawn based on just this sequence. In our study, p53 expression levels in lesions and corresponding rafts directly correlated with transformation levels, indicating that EcPV2 E6 might be unable to degrade p53. Alternatively, it is possible that EcPV2-infected cells stained positive for mutant (dysfunctional) p53 since p53 overexpression can be caused by an accumulation of mutant p53 [49,50]. Further studies are necessary to address this important issue.

## 5. Conclusions

In summary, we have established the first in vitro model for the study of EcPV2-associated equine tumor disease. We showed that raft cultures established from equine penile perilesional skin, plaques and SCC lesions accurately recapitulate major characteristics of the respective ex vivo tissue and, therefore, constitute a valuable tool for future investigation of carcinogenic factors driving EcPV2-associated disease onset and progression in the horse. 

## Figures and Tables

**Table 1:** Immunohistochemistry (IHC) antibody specifications.

Primary Antibody	Use	Retrieval	2nd Antibody	Chromogen	Control
Monoclonal mouse vimentin (M7020, Dako, Agilent, Santa Clara, CA, USA)	1:300, 30 min	98 °C, pH 6	MACH4 (BioCare Medical, Pacheco, CA, USA)	DAB	Equine skin
Monoclonal mouse PCK-26 (NB120-6401; Novus Biologicals LLC, Boulder, CO, USA)	1:500, 60 min	98 °C, pH 9, blocking serum	EnVision Mouse (K4001, Dako, Agilent, Santa Clara, CA, USA)	DAB	Equine skin
Monoclonal mouse anti-p53 (clone DO1; Santa CruzBiotechnology Inc, Santa Cruz, CA, USA)	1:100, 60 min	98 °C; pH 9	EnVision Mouse (K4001; Dako, Agilent, Santa Clara, CA, USA)	DAB	Equine squmamous cell carcinoma
Monoclonal mouse anti-Ki67 (clone MIB-1; Dako, Agilent, Santa Clara, CA, USA)	1:50, 60 min	98 °C; pH 9	REAL Kit (K5001; Dako, Agilent, Santa Clara, CA, USA)	AEC	Equine skin
Monoclonal mouse anti-MCM7 (clone DCS-141.2; Santa CruzBiotechnology Inc, Santa Cruz, CA, USA)	1:400, 60 min	98 °C; pH 9	EnVision Mouse (K4001, Dako, Agilent, Santa Clara, CA, USA)	DAB	Equine lymph node

DAB: diaminobenzidine, AEC: 3-amino-9-ethylcarbazole.

**Table 2:** RNA in situ hybridization (RISH) analysis of EcPV2 E6/E7 mRNA in raft and tissue sections.

Sections from	GS	DNS	Figure
Perilesional skin-derived rafts	+/−	−	Figure 2A
Perilesional skin	+/−	−	
Plaque-derived rafts	+	+	Figure 2B
Plaque tissue	+	++	
SCC-derived rafts	++	+	Figure 2C
SCC tissue	++	+/−	

SCC: squamous cell carcinoma; GS: granular signal; DNS: diffuse nuclear signal; −: no signal; +/−: sporadic signal; + moderate signal; +++ strong signal.

## Data Availability

Data is contained within the article.
